# Protocol for Diagnostic Test Accuracy Study: Evaluation of Cone Beam Computed Tomography (CBCT) in Prediction of Inferior Alveolar Nerve Injury as Compared to Orthopantomography (OPG) Secondary to Surgical Removal of Impacted Mandibular Third Molars

**DOI:** 10.7759/cureus.66864

**Published:** 2024-08-14

**Authors:** Abdul Ahad G Khan, Rajiv Borle

**Affiliations:** 1 Department of Oral and Maxillofacial Surgery, Sharad Pawar Dental College and Hospital, Datta Meghe Institute of Higher Education and Research, Wardha, IND

**Keywords:** anesthesia, paresthesia, risk factors, inferior alveolar nerve injury, orthopantomogram, cone beam computed tomography, impacted mandibular third molars

## Abstract

Introduction

Impairment of the inferior alveolar/dental nerve (IAN) is a relatively uncommon complication after lower wisdom tooth removal. Studies report varying incidences of IAN injury, with dysesthesia being noted as particularly distressing and 0-0.9% cases extending for a long duration. Neurosensory disruptions can severely impact speech, chewing, swallowing, and social interactions, leading to chronic pain and a lower quality of life. It also poses a risk of inadvertent injuries during meals. Although orthopantomogram (OPG) is primarily used for diagnosis, but when the lower wisdom tooth and nerve are in close approximation, cone beam computed tomography (CBCT) is recommended, despite its higher cost and radiation exposure. A white paper on third molar management necessitates further research on CBCT's role, citing conflicting evidence. Further in a multicentric trial, the difference between the OPG versus CBCT group was not statistically significant due to the low incidence of IAN injuries. They have emphasized the need for more well-designed studies to reach a statistically significant conclusion by meta-analyses. Hence, this study aims to provide additional evidence.

Methods

It is a two-arm, parallel, diagnostic study design involving individuals between the ages of 18 and 50 years, requiring lower wisdom tooth removal that is closely approximated with the nerve. Eligible adults, based on the specified inclusion/exclusion criteria, will be recruited into the study; informed consent will be obtained; then assigned randomly to the OPG or CBCT group using a random computer-generated sequence. Extractions will be done under local anesthesia using a standard surgical protocol with odontectomy. Surgical variables will include the experience of the surgeon, amongst others. The outcome variables will be recorded using patient interviews (subjective) and objective examinations from day one up to six months after surgery. The primary outcome will comprise the number of patients reporting abnormal sensations post-surgery. Secondary outcomes will include objectively confirmed IAN injuries and permanent IAN injuries (>6 months). Results will be analyzed statistically to look for significance and possible risk factors associated with it.

Results

If a statistically significant result is obtained, then we can deliberately reduce CBCT referrals and reserve them only for high-risk cases, wherein the risk of IAN injury cannot be predicted by OPG alone. If the experience of the surgeon proves to be an important risk factor, then it can also help refer high-risk patients to surgeons with more experience.

Conclusion

If CBCT proves to be statistically superior to OPG in the prediction of nerve injury, then we will be able to avoid significant morbidity and improve the quality of life of such patients by either modifying the surgical steps or by choosing other conservative treatment modalities. Further, this may reduce unnecessary CBCT referrals, thus reducing radiation exposure, the cost to patients, and, in turn, national healthcare expenditure. Besides, CBCT is not available at all centers, so a lot of low-risk patients can be managed safely at primary health centers, thus reducing the urban patient load.

## Introduction

Impairment of the inferior dental nerve (IAN) is one of the hazards and anticipated post-surgical complications, consequent to the extraction of lower wisdom teeth. Padhye and colleagues (2013) studied data from 1200 individuals examined over a five-year period to conclude that impairment of the inferior dental nerve is the most dreadful complication after removal of lower wisdom teeth [1]. Their findings were indistinguishable with the findings of Susarla and colleagues and comparable to Blondeau and colleagues, with 1.1% cases of paresthesia, including 0.5% lasting for more than a year [2,3]. Gulicher and colleagues, along with Valmaseda and colleagues [4,5], discovered a 0.4-6% chance of temporary IAN injury following removal of the third molar and a less than 1% chance of chronic IAN injuries lasting longer than six months. Ziccardi and Zuniga (2007) discovered a 0.4-22% incidence of impairment of IAN and lingual nerve related to third molar extraction [6].

Goldberg and colleagues found that there was an overall complication rate of 7%, including dysesthesia rates of 1.2% (of which 0.2% were permanent) post mandibular third molar extraction. They also cited dysesthesia as arguably the most upsetting side effect after third molar surgery [7]. In order to achieve an agreement on the best course of action for treating third molars, Haug and colleagues (2009) reviewed evidence-based resources and identified inferior alveolar nerve injury as a grave problem with a persistent involvement of 0-0.9% (> 6 months duration) [8].

Patients find it challenging to tolerate or acclimatize to neurosensory disruptions because of the high density of peripheral nerve receptors in the face and perioral area. These injuries can have crippling effects on a person's ability to talk, chew, swallow, and interact with others in social situations. There is also a possibility of inadvertent injuries, including burns and bites, because of the absence of sensation. Moreover, some injuries may leave behind permanent damage, ranging from mild hypoesthesia to entire paresthesia, or even neuropathic reactions that result in chronic pain syndromes [6]. Leung discovered that the affected person's quality of life was worse than that of normal people; they experienced more depressive symptoms, and their overall satisfaction with life was lower than that of normal people [9].

An orthopantomogram (OPG) is established diagnostic equipment that is used to assess the mandibular wisdom teeth and their relationship to the inferior dental nerve before surgery [10]. But it is a two-dimensional imaging, representing a three-dimensional object. There are some radiographic techniques, such as the cone shift technique, that can be helpful in ascertaining the association of the lower wisdom tooth root with the inferior dental nerve, but it warrants additional radiation exposure. A literature review has identified different radiographic signs that indicate nearness of the lower wisdom tooth root to the inferior dental nerve [11]. If OPG shows any of the radiographic signs of close approximation, then it is recommended to get 3DCT or cone beam computed tomography (CBCT) done to gather more information in the third dimension as well.

CBCT is being widely used nowadays in pre-treatment evaluation of mandibular third molars. But it is more expensive, not available at all health care centers, and has much more radiation exposure (102 microSv for the mandible alone) as compared to OPG (24 microSv) [12,13]. So it is important to weigh the benefits versus potential risks. An abstract from the conclusions of the white paper on lower wisdom tooth data denotes that the purpose of CBCT in the management of mandibular wisdom teeth is ambiguous and developing and urges further research to clarify the conditions for their practical application [10].

There has been conflicting evidence on the comparative analysis of CBCT and OPG in anticipating inferior dental nerve exposure during lower wisdom tooth extraction [14,15,16]. A study utilizing data from insurance companies did not find any reduction in the incidence of long-term impairment of IAN, despite an increase in CBCT usage [17]. A multicentric randomized trial was aimed at exploring the efficacy of CBCT to OPG on the possibility of temporary inferior dental nerve injury after extraction of lower wisdom teeth. In this trial, the two groups did not differ statistically [18]. The reduced incidence of IAN injury dictates the need for more similar well-designed studies to proceed with meta-analyses to reach meaningful conclusions. Hence, this study is designed so as to contribute additional evidence.

Study aim

To evaluate the usefulness of preoperative cone beam computed tomography in the prediction of impairment of inferior alveolar nerve in impacted mandibular third molar surgery as compared to orthopantomography.

Objectives

The three objectives of the study were to correlate the findings of OPG/CBCT with surgical outcomes in the prediction of inferior alveolar nerve injury in impacted mandibular third molar surgery; to delineate the risk factors responsible for impairment of inferior alveolar nerve in mandibular third molar surgery, and to generate a protocol for CBCT referrals in close approximation cases.

Clinical trial registration

The trial is registered with the Clinical Trials Registry of India with reference no CTRI/2023/05/052757.

## Materials and methods

Study design and population

It is a parallel-arm, diagnostic study design. The study population will include individuals between 18 and 50 years of age, consulting for mandibular third molar removal, that have an intimate relationship with inferior dental nerve.

Clinical and radiographic inclusion criteria

Healthy patients or those with controlled co-morbid conditions who are willing to undergo surgery for removal of impacted mandibular wisdom teeth in the age group of 18 to 50 years will be included in the study. Radiographic inclusion criteria include a close approximation of the inferior dental canal to roots of mandibular wisdom teeth, as evidenced by the observance of one or more of the following signs, visible on OPG, namely darkening of the root, narrowing of the canal, dark and bifid root, diversion of the mandibular canal, deflection of the roots, interruption of the white line of the mandibular canal wall, and narrowing of the roots [11].

Clinical and radiographic exclusion criteria

Patients with uncontrolled co-morbidities (American Society of Anesthesiologists (ASA) 3 or higher), a history of nerve disorders or diseases affecting nerves (such as neuralgia or stroke), any existing pathology (like cyst or tumor), previous CBCT scan, or those not willing to undergo surgery for removing impacted lower wisdom teeth will be excluded from the study. Pregnant patients will also be excluded. Patients with impacted lower wisdom teeth that are not closely associated with the inferior alveolar neurovascular bundle will comprise radiographic exclusion criteria.

Participant recruitment

Adults (between 18 and 50 years) with one or two lower wisdom teeth in close approximation to the inferior dental canal (as observed on OPG) following the above stated clinical and radiographic inclusion/exclusion criteria shall be incorporated into the study. The patients will be intimated about the course of the study, the possible consequences, the further line of management, and their informed consent will be taken. The patients will be recruited and randomly assigned to either of the two groups (OPG group and CBCT group) using a random computer generation sequence. The allocation concealment will be ascertained by contacting the principal investigator for group allocation.

**Figure 1:**
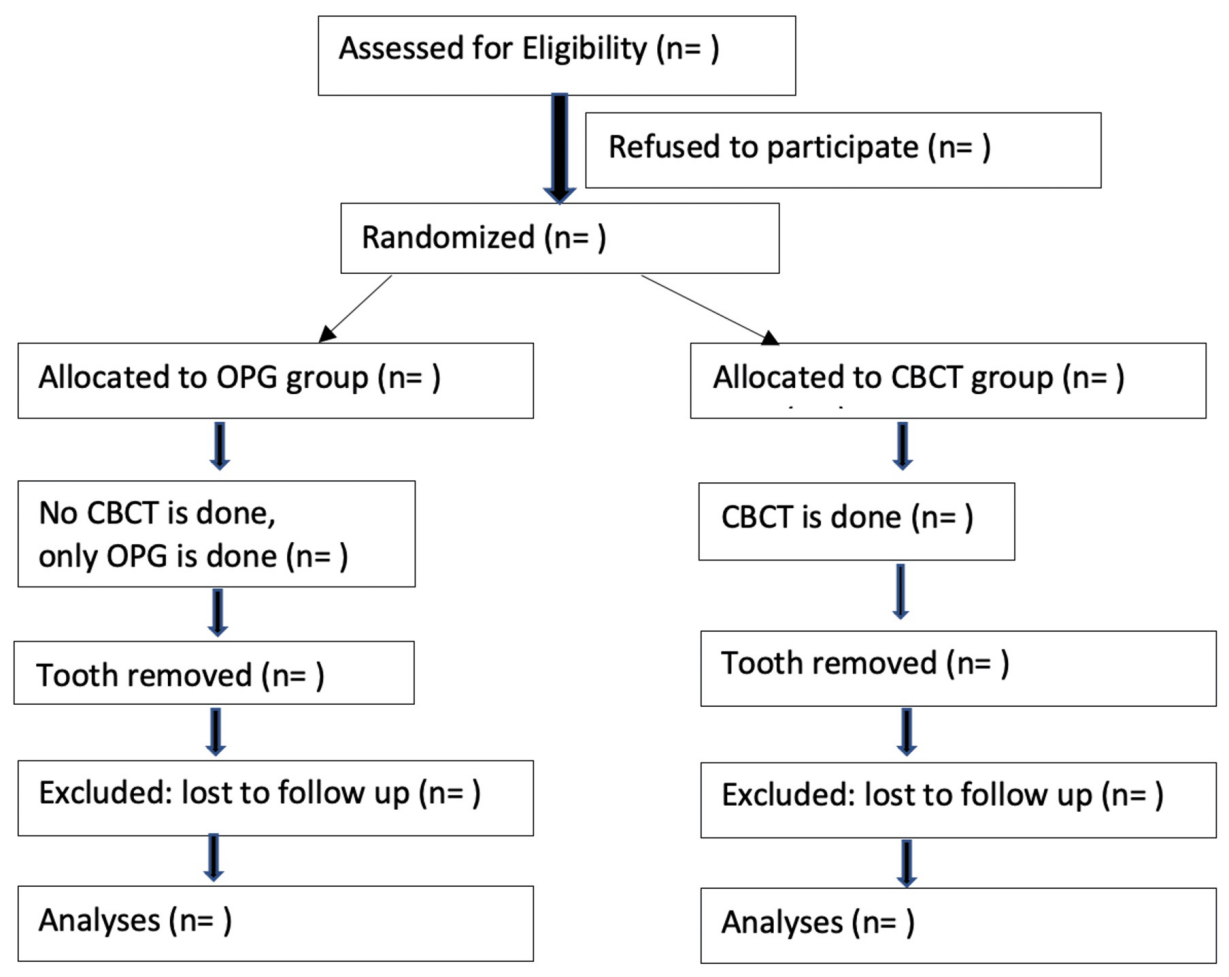
CONSORT flow diagram [19]

In the OPG group, the patients will be subjected to digital OPG alone (Planmeca Promax-Planmeca, Helsinki, Finland). In the CBCT group, a high-resolution CBCT scan will be done for the mandible preoperatively in addition to baseline OPG (Planmeca Promax 3D - Planmeca, Helsinki, Finland). CBCT/OPG images will be assessed by experienced surgeons. They will decide their surgical steps based on information from the OPG/CBCT images. All lower wisdom teeth will be removed using local anesthesia by standard surgical protocol with odontectomy. Variables, including the operating surgeon’s experience, will be noted down along with the outcome variables of subjective and objective nerve injury from day one till six months after surgery. The CONSORT flow diagram showing the progress of the study is outlined in Figure 1.

Reference standard and its rationale

Orthopantomogram (OPG) is established diagnostic equipment that is used to assess the lower wisdom teeth and their relationship with the inferior dental nerve canal before surgery [10]. Due to challenges associated with the placement of the intraoral receptor during periapical radiography, for evaluating mandibular third molars, it is recommended to employ OPG as a primary imaging modality prior to surgical extraction. OPG is advantageous, as it can be conducted with minimal patient discomfort compared to intraoral techniques. Additionally, it is a low-radiation method that captures all wisdom teeth in a single film, equivalent to approximately two to 16 intraoral film exposures.

Patient screening process

In the assessment of OPG, the initial step involves determining if there is observance of one or more of Rood and Shehab’s criteria indicating close approximation between the root of the third molar and inferior alveolar canal. This will be the eligibility criteria for patient recruitment. All these patients will be assigned to either the OPG group or the CBCT group by random computer-generated sequence. Group allocation will be done by contacting the principal investigator for randomization. In the OPG group, patients will not be subjected to any other radiographs, whereas in the CBCT group, a CBCT will be done for the mandible in addition to the digital OPG used for screening. This is done to more accurately understand the spatial association between lower wisdom tooth roots and inferior dental canal. The OPG will be assessed for variables such as angulation, depth, difficulty index, length of red line, Rood Shehab’s criteria, as well as type of vertical overlap of third molar root with inferior alveolar canal [11,20].

CBCT images will be evaluated in all three planes. The images will be evaluated to find out the distance from the lower wisdom tooth root to the inferior dental canal and the continuity of the cortical layer between the neurovascular bundle and root. The spatial orientation of the inferior dental canal with regard to the wisdom tooth root will be noted down as either buccal, lingual, inferior, or interradicular. The shape of the IAC at the exact place of contact with the wisdom tooth root will be noted down as oval, round, or narrowed.

The investigator assessing the primary and secondary outcomes will be blinded to the groups. The number of patients reporting subjective symptoms of inferior alveolar nerve injury after surgery will be considered as the primary outcome. The secondary outcome will include the number of patients experiencing signs and symptoms of nerve injury that are confirmed by an abnormal clinical neurosensory test. It will also include those patients having symptoms of injury lasting more than six months post-surgery. First, the patients will be enquired about abnormal sensations in the ipsilateral lip and chin and compared to the contralateral side. This will be confirmed by standard clinical quantitative nerve testing procedures such as static light touch, two-point discrimination, brush-directional discrimination, and pin pressure nociception [21]. Patients with altered sensation will be examined at day one, day seven, day 30, day 60, day 90, and then day 180 postoperatively to assess their recovery pattern. If needed, further consultations will be taken for persistent paresthesia/anesthesia.

Training and calibration of personnel

Trained oral and maxillofacial surgeons will carry out the assessment independently. They will be experienced in the identification and diagnosis of maxillofacial structures utilizing both OPG and CBCT. They will be blinded by the clinical outcome. All three planes will be assessed through the implant planning screen and multiplanar reconstruction screen.

Mitigation of confounders

There can be various confounders in this study. First of all, the patients will be randomized to the two groups based on a random computer generator sequence to avoid selection bias. Group allocation will be done by contacting the principal investigator for randomization. Experienced surgeons will be carrying out the procedure, and their experience will be noted down. Besides, it will be performed by different surgeons for external validation. The type of impaction will be noted to assess difficulty. The technique of surgical removal will be standard, involving odontectomy. The evaluator of the outcome will be blinded to the group of study participants.

Sample size calculation

Hypotheses will be tested using the one-tailed superiority analysis method, with 80% power and a 5% level of significance, based on the incidence of patient-reported abnormal sensations after surgery. Considering a superiority margin of 33% as per reference values between the incidence of temporary inferior dental nerve paresthesia of 25% in the OPG group and 58% in the CBCT group, the total sample size will be 68 (34 in each group) using the following formula [18].

 

$$p=(p_1+rp_2)/(1+r )$$

$$n &ge; [Z_(1-&alpha;/2) &radic;((r+1)p(1-p) )+Z_(1-&beta;) &radic;(rp_1 (1-p_1 )+p_2 (1-p_2 ) )]^2/(r (p_2-p_1 )^2 )$$

## Results

The results will be described based on Standards for Reporting of Diagnostic Accuracy (STARD) guidelines. The statistical analyses will be done with the ‘intention to treat’ principle. The complete dataset analysis will encompass all study participants, based on the mentioned inclusion/exclusion criteria, who have no missing data for any of the parameters in the dataset. The primary outcome will include the number of patients experiencing inferior dental nerve injury as evidenced by the subjective presence of paresthesia, anesthesia, or dysesthesia after mandibular third molar surgery. Secondary outcomes will include the number of patients with nerve injury confirmed by abnormal clinical neurosensory testing and nerve injury lasting for more than six months.

Baseline characteristics will be used to describe data. Mean and standard deviation will be used to describe continuous variables. Frequencies and percentages will be used to describe categorical data. The outcome variables for continuous data, if normally distributed, will be summarized by using the mean, range, standard error, standard deviation, and confidence interval at 95%. The outcome variables for continuous data will be analyzed using an independent sample t test at the 5% level of significance (P<=0.05) assuming a normal distribution.

Data not following normal distribution will be described by nonparametric tests using mean, median, lower, and upper quartiles. A Mann-Whitney test will be used for testing such significance. Chi square analysis will be utilized to analyze efficacy for the categorical variables. Fisher exact or chi-square test will be used to analyze nominal and dichotomous data. Analysis for logistic regression will be done to recognize possible risk factors for impairment of the inferior alveolar nerve. Comparative groups will be analyzed by a t test at the 5% level of significance (P<=0.05). Sensitivity and specificity of CBCT and OPG in prediction of nerve injury will be confirmed on the basis of results obtained on subjective/objective nerve testing after surgical removal of a tooth (Table 1).

**Table 1:** Master chart format for recording various study variables

	Demographic variables									Pathological variables			OPG findings							Cone beam computed tomography					Surgical variables							outcome variables													
Patient	Age	Sex	ASA class	Medical co-morbidity	Smoking (frequency)	Alcohol (frequency)	Oral contraceptive use (Y/N)	Oral hygiene (good, moderate. Poor)	Side (38/48)	Pericoronitis (Y/N)	Pocket >4mm(Y/N)	Caries(Y/)	Angulation	Depth of impaction (A, B,C)	Ramus overlap (I, II,III)	Difficulty index	Length of red line (in mm)	Rood Shehab criteria (name/s)	Vertical over projection of root with inf alv canal Class 1,2,3,>3	Inf alv canal position in relation to root (inf, ling, buccal, inter-radicular)	Distance between root and IA canal vertically and buccolingually	Root in contact with IA canal (Y/N)	Cortical layer of canal (present/absent)	Shape of canal (round/oval/narrowing)	Experience of surgeon (<100 M3 removed, 101-500 M3 removed, 501-1000 M3 removed, 1001-3000 M3 removed, >3000 M3 removed)	Incision/flap (envelope/triangular/quadrilateral)	Bone removal (Y/N)	Odontectomy (Y/N)	No.of roots (1,2,>2)	Inf alv nerve visible (Y/N)	Intra-operative hemorrhage from IA canal	Subjective inf alv nerve injury (Y/N)						objective inf alv nerve injury (Y/N)						infection	any other complication
																																Day 1	Day 7	1 month	2 months	3 months	6 months	Day 1	Day 7	1 month	2 months	3 months	6 months		
1																																													
2																																													
3																																													
4																																													
5																																													
6																																													
7																																													
8																																													
9																																													
10																																													

## Discussion

This study investigates the clinical utility of cone beam computed tomography compared to orthopantomography in predicting inferior alveolar nerve injury during impacted mandibular third molar surgery. While CBCT offers superior anatomical visualization, its impact on patient outcomes and surgical decision-making requires further clarification.

This prospective study employs a robust methodology, including standardized surgical techniques and blinded outcome assessments, to minimize bias and maximize reliability. Comprehensive statistical analysis will be used to compare the diagnostic accuracy of CBCT and OPG in predicting IAN impairment and identify associated risk factors.

The study's strengths lie in its prospective design and rigorous methodology. However, limitations include potential generalizability constraints due to the subjective nature of some outcome measures.

This study is anticipated to generate valuable data regarding the comparative effectiveness of CBCT and OPG in predicting IAN injury. By correlating imaging findings with surgical outcomes and identifying risk factors, the study aims to guide clinicians in preoperative imaging decisions and surgical planning. Developing a clear protocol for CBCT referrals in close approximation cases can optimize resource allocation, minimize radiation exposure, and improve patient care.

This study holds significant clinical relevance by addressing a common surgical challenge and aiming to improve patient outcomes. The findings have the potential to inform clinical practice guidelines, enhance preoperative risk assessment, and contribute to safer and more predictable outcomes in impacted mandibular third molar surgery.

## Conclusions

A statistically significant result will help generate evidence for the reduction of CBCT referrals to high-risk cases, wherein OPG fails to adequately assess IAN injury risk. Such a strategy would reduce unnecessary radiation exposure, patient costs, and also national healthcare expenditure. It will also enable identification and effective management of low-risk cases at primary health centers, thus diminishing urban patient overload. Moreover, if the surgeon's experience proves to be a significant predictor, then it may help guide the referral of high-risk patients to more experienced surgeons for tooth removal or to opt for other conservative procedures such as coronectomy.
